# Hydrochlorothiazide and risk of hearing disorder: a case series

**DOI:** 10.1186/s13256-018-1580-8

**Published:** 2018-05-20

**Authors:** Natnael Belai, Selamawit Gebrehiwet, Yodit Fitsum, Mulugeta Russom

**Affiliations:** 1 Orotta National Referral Hospital, Asmara, Eritrea; 2 Eritrean Pharmacovigilance Centre, Asmara, Eritrea; 3 Edaga-Hamus Community Hospital, Asmara, Eritrea

**Keywords:** Hydrochlorothiazide, Hearing disorder, WHO global adverse drug reaction database

## Abstract

**Background:**

Hydrochlorothiazide is not known to cause hearing disorder. The Eritrean Pharmacovigilance Centre, however, has received cases of hearing disorder, including irreversible deafness, associated with hydrochlorothiazide. The aim of this study is, therefore, to assess the causal relationship between hydrochlorothiazide and hearing disorder.

**Methods:**

Data was retrieved from the WHO global adverse drug reaction database, VigiBase™. A search on VigiBase™ was made on August 6, 2017 using “hydrochlorothiazide” as drug substance, and “ototoxicity”, “decreased hearing”, and “vestibular disorder” as reaction terms. Cases were retrieved using VigiLyze™ and exported to an Excel spreadsheet for descriptive analysis. Causality was assessed using Austin Bradford-Hill criteria and labeledness of the reaction was evaluated through a thorough literature review including the summary of product characteristics.

**Results:**

From 1972 to August 2017, 94 cases of hearing disorder associated with hydrochlorothiazide were submitted from 18 countries to VigiBase™. The median age was 64 years with almost equal male to female ratio. In 53.2% of the cases, hydrochlorothiazide was reported as the only suspected drug. Of these, 26 cases encountered hearing disorder following the sole intake of hydrochlorothiazide. Reaction was marked as “serious” in 36% of the cases and median time to reaction onset was 3 days. Outcome was reported as reversible in 66.7% of the cases. Reaction resolved in 17 cases following withdrawal of hydrochlorothiazide and recurred in one case after subsequent rechallenge with the product. Consistency of cases and a dose–response relationship was also observed in this case series.

**Conclusions:**

This case series assessment found a suggestive causal relationship between hydrochlorothiazide and hearing disorder. Taking the inherent limitations of this study into account, results should be interpreted with caution and further studies are required to validate the safety signal.

## Background

Hydrochlorothiazide is a thiazide diuretic that is used in the treatment of hypertension and edema either as the sole therapeutic agent or in combination with other drugs [1, 2]. To the best of our knowledge, there are no published articles or case reports that associate hydrochlorothiazide (HCTZ) and hearing disorder or ototoxicity. HCTZ has only been associated with vertigo and there are also reports of tinnitus when combined with amiloride [3]. The summary of product characteristics (SPC) of HCTZ found on the webpage of the European Medicines Agency (EMA) [4], Medicines and Healthcare Products Regulatory Agency (MHRA) [5], US Food and Drug Administration (FDA) [6] and others [7] do not mention ototoxicity or hearing disorder as an adverse effect. Ototoxicity is defined by the toxic capacity of certain drugs or toxins relative to the inner ear structures, particularly to the cochlea and the vestibular cells, or the acoustic nerve [8, 9]. The symptoms of cochleotoxicity range from mild tinnitus to total hearing loss and vestibulotoxicity may range from mild imbalance to total incapacitation [10].

Even though HCTZ is not known to cause hearing disorder, the Eritrean Pharmacovigilance Centre received two cases of hearing disorder, including irreversible deafness, associated with the use of HCTZ. The objective of this study is, therefore, to assess the causal association between HCTZ and hearing loss through data mining on the WHO global adverse drug reaction database, VigiBase™.

## Methods

Data was retrieved from the WHO global adverse drug reaction database, VigiBase™, which is developed and maintained by the Uppsala Monitoring Centre (UMC), Sweden. The database captures adverse drug reaction reports submitted from National Pharmacovigilance Centres of more than 120 countries. A search on VigiBase™ was made on August 6, 2017 using “hydrochlorothiazide” as drug substance, and “ototoxicity” [preferred term (PT)], “hearing decreased” [high-level term (HLT)], and “vestibular disorder” (HLT) as reaction terms [World Health Organization Adverse Reaction Terminology (WHO-ART)]. All relevant information like the reaction outcome, seriousness, information component (IC), outcome of rechallenge/dechallenge, and patients’ background characteristics were extracted using VigiLyze™, a tool developed by the UMC for data mining and analysis of adverse drug reactions (ADR) in VigiBase™. IC value is a measure of disproportionality of drug ADR in VigiBase™ [11, 12]. A positive IC value indicates that a particular drug ADR pair is reported more often than expected, based on all the reports in VigiBase™. The results generated were then exported to an Excel spreadsheet for descriptive analysis. Potential duplicate cases were eliminated using VigiMatch™, a tool developed by the UMC.

The series of cases were then subjected to Austin Bradford-Hill criteria [13] to assess the causal association between HCTZ and hearing disorder. Using Bradford-Hill criteria, strength of association, consistency of cases, specificity of the association, temporal relationship, dose–response relationship, biological plausibility, experimental evidence, coherence, and analogy of the association was assessed in case series. Expectedness or labeledness of the adverse effect was assessed through thorough literature review including the SPCs.

## Results

The Eritrean Pharmacovigilance Centre has received two cases of HCTZ-associated hearing disorder. In both cases, HCTZ was the sole suspect with no other concomitants. The causality assessment using Naranjo adverse drug reaction probability scale [14] was found to be “probable” for both cases.

### Case presentation

#### Case 1

A 50-year-old hypertensive woman, 75 kg, Tigrigna ethnic group, was prescribed with enalapril 20 mg two times a day and hydrochlorothiazide 25 mg three times a day for the management of hypertension. Upon taking the medications, a pharmacist told her to take two times enalapril 20 mg and three times hydrochlorothiazide 25 mg, overall five tablets a day. The poor lady mistakenly understood the five tablets a day as “five times a day” and for that she took hydrochlorothiazide 25 mg five times a day for 3 days starting from June 10, 2013. She experienced loss of balance and dizziness on the first day and complete hearing loss on the third day following the intake of HCTZ. The patient was followed for 6 months and despite dose reduction, her condition was irreversible.

#### Case 2

A 65-year-old man, Bilen ethnic group, experienced hearing loss, epigastric discomfort, and nausea following the intake of oral hydrochlorothiazide 25 mg two times a day for mild hypertension on May 5, 2015. The reaction (hearing loss) started the same day as the intake of HCTZ and started to resolve 1 day after discontinuation of the suspected drug.

From 1972 to August 2017, a total of 94 cases of hearing disorder associated with HCTZ were submitted from 18 countries to the VigiBase™. The reactions reported were tinnitus (50), deafness (23), decreased hearing (16), vestibular disorder (4), and deafness nerve (1). Age was reported in 72 (77%) of the cases and around 47% of them were between 45 and 74 years with a median age of 64 (Table 1). Sex was also reported in all cases with almost equal male to female ratio (Table 1).

**Table 1:** Distribution of the cases according to background characteristics

Background characteristics	N (%)
Age at index date (years)	
Less than 45	04 (4.3)
45–59	25 (26.6)
60 and above	43 (45.7)
Unknown	22 (23.4)
Sex	
Male	48 (51.06)
Female	46 (48.93)

Time to reaction onset was reported in 34 cases and the median onset was found to be 3 days. In 55 of the cases, daily dose was reported within the normal therapeutic dose (12.5–50 mg per day). In 17 of the cases, reaction abated following withdrawal of HCTZ and in one case reaction recurred after reintroduction of HCTZ (Table 2).

**Table 2:** Cases of hearing disorder associated with hydrochlorothiazide retrieved from VigiBase™

	Reaction term	Cases retrieved	Cases with single drug suspect	Cases with positive dechallenge	Cases with positive rechallenge
1.	Tinnitus	50	29	14	1
2.	Deafness	23	15	1	0
3.	Hearing decreased	16	6	2	0
4.	Vestibular disorder	4	2	0	0
5.	Deafness nerve	1	0	0	0
	Total	94	53^a^	17	1

^a^In three cases patients manifest more than one reaction, which makes it 53 instead of 50

In 50 of the cases, HCTZ was reported as the only suspected drug and in 26 cases HCTZ was found to be the sole suspected drug with no other concomitants (Table 2). The most frequently co-reported adverse reactions in order were dizziness, headache, dyspnea, fatigue, and hypertension, occurring in 10–20% of the cases. Amlodipine (4.6%), acetylsalicylic acid (3.9%), metoprolol (3.3%), losartan (2.6%), and levothyroxine (2.6%) were among the co-reported drugs with HCTZ.

Seriousness of the reaction was reported in 34 of the cases and 36% of them were marked as “serious”. In 17 cases, reaction resolved following withdrawal of HCTZ and reaction recurred in one case following reintroduction of HCTZ (Table 2). Reaction outcome was reported as “recovered”/“recovering” in 33% of the cases and “not yet recovered” in 17%. Outcome is unknown in the rest of the cases.

## Discussion

In this case series assessment, the relationship of HCTZ and hearing disorder was assessed using Austin Bradford-Hill criteria (Table 3). The wide geographic distribution of the case reports, the plausible time relationship, the positive dechallenge and rechallenge reported in some cases, cases with hearing loss manifested on the second day following administration of HCTZ with no concomitants, and the irreversible deafness observed shortly after overdose intake of HCTZ supports a causal association.

**Table 3:** Results of causality assessment on hydrochlorothiazide and hearing disorder using Austin Bradford-Hill criteria

	Criterion	Outcome
1.	Strength of association	IC value is negative (no statistical signal).
2.	Consistency of the cases	In many of the cases, reaction manifested within a few days (between 1 and 7 days) following the commencement of HCTZ. The cases have wide geographical distribution with similar clinical features that ranges from tinnitus to deafness shortly following administration of HCTZ.
3.	Specificity of the association	In 26 of the cases, HCTZ was the only suspected drug with no other concomitants and patients experienced reactions with similar clinical features like tinnitus, ototoxicity, hearing loss, and/or deafness. Nevertheless, the availability of multiple co-reported drugs and reactions in many of the other cases make the association nonspecific.
4.	Temporal relationship	All reactions manifested after HCTZ was administered with median time to reaction onset of 3 days. In six cases, hearing loss manifested on the second day following administration of only HCTZ.
5.	Dose–response relationship	In the case reported from Eritrea, a patient experienced irreversible deafness within 3 days after taking an overdose of HCTZ (25 mg five times a day for 3 days).
6.	Biological mechanism or plausibility	There is no clear biological mechanism by which HCTZ causes hearing loss. However, medications affecting sodium and potassium transport in kidney tissues alter ionic homeostasis of the inner ear causing functional problems like hearing loss, tinnitus and vertigo. This is evidenced by inner ear tissues being immunologically, biochemically, and functionally related to kidney tissues [19].
7.	Experimental evidence	No evidence found in animal studies. However, the positive dechallenge and rechallenge supports the association.
8.	Analogy	There are also reports of tinnitus in the combination product, amiloride and HCTZ, though tinnitus is not yet associated with HCTZ. Loop diuretics are also known to cause hearing disorder through different mechanism of action.
9.	Coherence	Not applicable

*IC* information component, *HCTZ* hydrochlorothiazide

Although the availability of multiple co-reported drugs and reactions in many of the cases make the association nonspecific, in 28% of the cases, HCTZ was reported as the only suspected drug with no other concomitants.

Even though drug-induced hearing disorder is dose and duration related [15], 75% of the cases were manifested at normal therapeutic daily dose (12.5–50 mg/day) a few days after intake of HCTZ. This suggests that HCTZ-related hearing loss can occur even at normal dose; especially when administered with other ototoxic drugs.

It should, however, be noted that the association of HCTZ and hearing disorder can be affected by many confounders like age, the disease itself (hypertension), and other drugs taken concomitantly like acetylsalicylic acid (ASA) [16]. As the median age of the cases was 64 years, hearing disorder could be assumed as age related. On the other hand, age-related hearing loss is characterized by progressive (irreversible), symmetrical, and sensory neural hearing loss [17]; thus reversibility of the cases in 66.7% shows that the effect of age was not remarkable. Even though hypertension is known to cause sensory neural hearing loss [18], which usually is irreversible, the effect of the disease seems insignificant as outcome was reversible in the majority of the cases. ASA is also well known to cause reversible hearing loss, but the number of patients taking ASA in our study was small (3.9%); suggesting a minimal potential contribution. Similarly, the effect of other concurrently used drugs could not be ruled out due to the nature of the study. The effect of heredity, environmental or occupational hazards like noise pollution were also left as residual confounders. Taking into account the inherent limitation of spontaneous reporting, similar cases might be underreported, which can understate the association. Another limitation of the study is that, as we had no denominator data, the incidence of the potential risk cannot be estimated.

## Conclusions

From this case series assessment, it can be concluded that there is a suggestive causal association between HCTZ and hearing disorder. As it may cause irreversible hearing loss, this association warrants further study to substantiate the safety signal through epidemiologic studies. Taking into consideration the impact of hearing loss, difficulty in communication, loss of balance, incapacity, dependency and accidents, healthcare professionals should be cautious in prescribing ototoxic drugs concomitantly with HCTZ. Besides, they should also advise patients to avoid or minimize over-the-counter drugs, which are believed to be ototoxic like NSAIDS. We also recommend healthcare professionals to do routine hearing assessment for patients taking HCTZ.

## Abbreviations

ADR: Adverse drug reaction
ASA: Acetylsalicylic acid
EMA: European Medicines Agency
HCTZ: Hydrochlorothiazide
HLT: High-level term
IC: Information Component
MHRA: Medicines and Healthcare Products Regulatory Authority
PT: Preferred term
SPC: Summary of product characteristics
US FDA: United States Food and Drug Administration
WHO-ART: World Health Organization Adverse Reaction Terminology
